# Modular Drug-Loaded Nanocapsules with Metal Dome Layers as a Platform for Obtaining Synergistic Therapeutic Biological Activities

**DOI:** 10.1021/acsami.3c07188

**Published:** 2023-10-20

**Authors:** Arnon Fluksman, Aritz Lafuente, Ron Braunstein, Eliana Steinberg, Nethanel Friedman, Zhanna Yekhin, Alejandro G. Roca, Josep Nogues, Ronen Hazan, Borja Sepulveda, Ofra Benny

**Affiliations:** † Institute for Drug Research (IDR), School of Pharmacy, Faculty of Medicine, The Hebrew University of Jerusalem, 9112102 Jerusalem, Israel; ‡ Catalan Institute of Nanoscience and Nanotechnology (ICN2), CSIC and BIST, Campus UAB, 08193 Bellaterra, Barcelona, Spain; § Universitat Autònoma de Barcelona, Campus UAB, 08193 Cerdanyola del Vallès, Barcelona, Spain; ∥ Institute of Biomedical and Oral Research (IBOR), Faculty of Dental Medicine, The Hebrew University of Jerusalem, 9112102 Jerusalem, Israel; ⊥ Department of Bone Marrow Transplantation and Cancer Immunotherapy, Hadassah Medical Center, The Faculty of Medicine, The Hebrew University of Jerusalem, 9112102 Jerusalem, Israel; # ICREA, Pg. Lluís Companys 23, 08010 Barcelona, Spain; ∇ Instituto de Microelectronica de Barcelona (IMB-CNM, CSIC), Campus UAB, 08193 Bellaterra, Barcelona, Spain

**Keywords:** drug delivery, nanocapsules, multifunctional nanocapsules, Janus metal−polymer nanocapsules

## Abstract

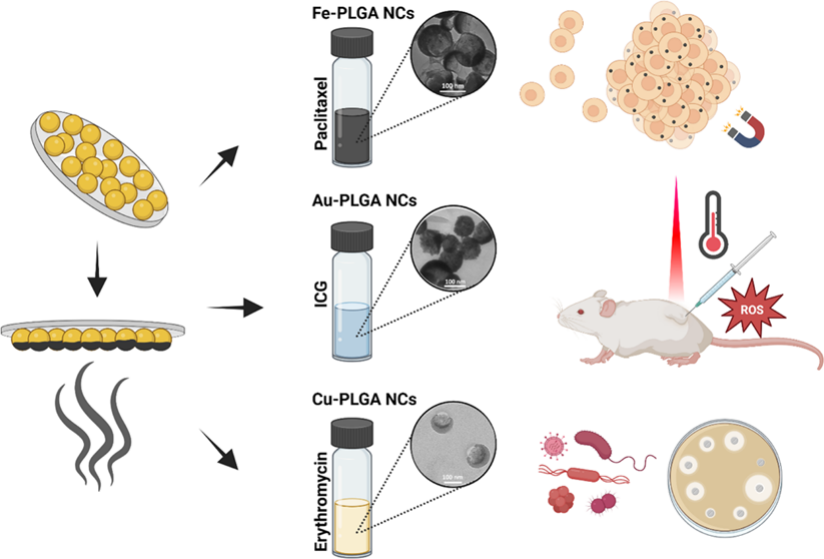

Multifunctional drug-loaded polymer–metal nanocapsules have attracted increasing attention in drug delivery due to their multifunctional potential endowed by drug activity and response to physicochemical stimuli. Current chemical synthesis methods of polymer/metal capsules require specific optimization of the different components to produce particles with precise properties, being particularly complex for Janus structures combining polymers and ferromagnetic and highly reactive metals. With the aim to generate tunable synergistic nanotherapeutic actuation with enhanced drug effects, here we demonstrate a versatile hybrid chemical/physical fabrication strategy to incorporate different functional metals with tailored magnetic, optical, or chemical properties on solid drug-loaded polymer nanoparticles. As archetypical examples, we present poly(lactic-*co*-glycolic acid) (PLGA) nanoparticles (diameters 100–150 nm) loaded with paclitaxel, indocyanine green, or erythromycin that are half-capped by either Fe, Au, or Cu layers, respectively, with application in three biomedical models. The Fe coating on paclitaxel-loaded nanocapsules permitted efficient magnetic enhancement of the cancer spheroid assembly, with 40% reduction of the cross-section area after 24 h, as well as a higher paclitaxel effect. In addition, the Fe-PLGA nanocapsules enabled external contactless manipulation of multicellular cancer spheroids with a speed of 150 μm/s. The Au-coated and indocyanine green-loaded nanocapsules demonstrated theranostic potential and enhanced anticancer activity in vitro and in vivo due to noninvasive fluorescence imaging with long penetration near-infrared (NIR) light and simultaneous photothermal–photodynamic actuation, showing a 3.5-fold reduction in the tumor volume growth with only 5 min of NIR illumination. Finally, the Cu-coated erythromycin-loaded nanocapsules exhibited enhanced antibacterial activity with a 2.5-fold reduction in the MIC50 concentration with respect to the free or encapsulated drug. Altogether, this technology can extend a nearly unlimited combination of metals, polymers, and drugs, thus enabling the integration of magnetic, optical, and electrochemical properties in drug-loaded nanoparticles to externally control and improve a wide range of biomedical applications.

## 1 Introduction

The advances in nanotechnology have generated a flourishing field of nanoparticulate systems for therapeutic and diagnostic applications.^1^ A wide diversity of materials has been used to formulate nanoparticles (NPs), including polymers, lipids, metals, carbon, and inorganic nonmetallic solids.^2^ NPs are used in drug delivery to stabilize the active compounds, enhance the circulation time of the encapsulated drugs, and improve the selectivity and tissue targeting. The improved pharmacological properties eventually lead to an enhanced therapeutic index while minimizing the off-target effects.^3,4^

In recent years, multifunctional platforms for drug delivery have been developed by combining one or more materials with different properties, thus providing new possibilities usually not available when used separately.^5−9^ In this regard, drug-loaded nanocapsules (NCs) comprising solid polymers with different metals have been extensively studied. Common fabrication methods are based on the coencapsulation of the polymer and the metal NP-containing solutions. The obtained products using these methods are typically isotropic, in the form of nanocapsules with metallic cores that are shelled by a polymeric layer or, alternatively, polymeric nanocapsules conjugated with metallic NPs on their surface, resulting in “patchy” nanocapsules or core–satellite nanocapsules.^10−13^ Metals such as iron, gold, palladium, silver, copper, and titanium were shown to be promising candidates for the functionalization of drug carriers by exploiting the biological, biochemical, or physical properties of the metals and polymers.^11,14−22^ However, these core–shell systems have substantial drawbacks, such as limited drug loading, metal masking by the polymeric shell which often limits their physicochemical response, and unpredictable release kinetics from polymer matrices.^11,14,17^ Another significant drawback in chemical synthesis methods that combine inorganic phases (metals or metal oxides) and organic phases (polymers) is the need to optimize each formulation element separately, which can be a time-consuming and complex process. Furthermore, if changes to the inorganic component are required, it often demands an entirely new fabrication process, adding further complexity and potential inefficiencies to the overall procedure.^12^ In the case of anisotropic Janus metal/polymer nanoparticles, the chemical synthesis processes are even more complex. The most common methods to chemically synthesize Janus particles include: (i) interfacial or emulsion polymerization,^23^ although they are mainly restricted to Janus polymer structures; (ii) seed-mediated growth,^24^ which involves the controlled growth of metal nanoparticles on preformed polymer particles or vice versa; and (iii) pickering emulsion^25^ using metal nanoparticles as emulsifiers to stabilize emulsions of two immiscible liquids, leading to the formation of polymer forms around the metal nanoparticles. These processes are even more complex when they involve the incorporation of a third component, as the drug, inside the polymer particle.

As an alternative, Janus metal/polymer nanostructures can be fabricated by adapting physical vapor deposition techniques typically used in the semiconductor, optic, aerospace, and solar industries for metal depositions,^26,27^ to deposit nanodome-shaped metal coatings on the polymer nanoparticles, i.e., polymer nanoparticles half-coated by metal layers. Such nanoparticles have shown some promising biomedical applications.^5^ For example, polystyrene, poly(acrylic acid), and silica templates were fabricated with metals for different purposes, such as cell targeting and biosensing,^28^ ultrasound and magnet propulsion,^29^ drug delivery, photothermal therapy,^30^ opto-magnetic nanoheating/thermometry,^31^ and theranostic applications.^32^ Nevertheless, the application of drug-loaded biodegradable polymer nanoparticles as templates for the development of active multiactuated nanocapsules is currently in its early stages of exploration. Interestingly, a recent work has demonstrated their potential in magnetically amplified chemo/photothermal cancer therapy,^33^ indicating a promising direction for future advancements in this area. However, there is still a need to demonstrate the capacity to extend this modular nanotherapeutic concept to other drugs and metals to achieve synergistic therapeutic effects in other biomedical applications.

Here, we present a tunable and robust strategy to fabricate multiple combinations of metal coating on drug-loaded nanocapsules as templates to achieve synergistic enhanced therapeutic effects. This fabrication strategy involves the self-assembly of drug-loaded nanocapsules in a solid support and the deposition of the different metal layers by physical vapor deposition. Therefore, once the drug-loaded polymer nanocapsule is optimized and self-assembled, any metal layer or multilayer can be deposited without the need for any further optimization process. To demonstrate the wide versatility and applicability of this polymer/metal nanostructure, we show the control of both the active encapsulated substance and the deposited metal. This enables the integration layers with customized magnetic, optical, and chemical properties to enhance the drug effects. As proof of concept, we selected a biodegradable and biocompatible copolymer, poly(lactic-*co*-glycolic acid) (PLGA),^34^ loaded with three different drugs and coated with three different metals to synergistically boost their therapeutic effects. We first show ferromagnetic iron-capped carriers (Fe-PLGA NCs) loaded with the drug paclitaxel, which enable the enhancment of assembly and external, contactless control of multicellular spheroids by using magnetic forces as well as increasing the efficacy of the loaded drug in the cancer spheroids. In our second demonstration, we show plasmonic gold-coated carriers (Au-PLGA NCs) loaded with indocyanine green (ICG) for theranostic application, enabling noninvasive imaging and simultaneous photothermal–photodynamic therapies with near-infrared (NIR) light. Finally, we demonstrate antibacterial copper-coated carriers (Cu-PLGA NCs) loaded with the antibiotic erythromycin, showing a synergistic antibacterial effect resulting from the release of copper cations. Altogether, our examples illustrate the capacity to easily integrate any active metal layer in drug-loaded polymer nanoparticles without perturbing the activity and release of the drugs. This integration serves to significantly amplify the drug effects, thereby showcasing its potential across a broad spectrum of biomedical applications.

## 2 Materials and Methods

### 2.1 Materials

Acid-terminated poly(d,l-lactic-*co*-glycolic acid) (PLGA) (RG502H 7000–17 000 Da, viscosity: 0.16–0.24 dL/g, 0.1% (w/v) in chloroform), poly(allylamine hydrochloride) (PAH), poly(diallyldimethylammonium chloride) (PDDA), (3-aminopropyl)trimethoxysilane (APTMS), poly(sodium 4-styrenesulfonate) (PSS), agarose, poly(vinyl alcohol) (PVA), coumarin-6, rhodamine 6G, Dulbecco’s phosphate-buffered solution (PBS), 2′,7′-dichlorofluorescein diacetate (DCFH-DA), and Dulbecco’s modified Eagle’s medium (DMEM) were purchased from Sigma-Aldrich (MO). Tween 80 was obtained from Fisher BioReagent (NJ). Solutol (Kolliphor HS 15) and erythromycin were purchased from Glentham (U.K.), and indocyanine green (ICG) was obtained from Chem-Impex (IL). Thiazolyl blue tetrazolium bromide (MTT) was purchased from Alfa Aesar (Lancashire, U.K.).

### 2.2 Preparation of Metal-Deposited Polymeric NPs

**Scheme 1:**
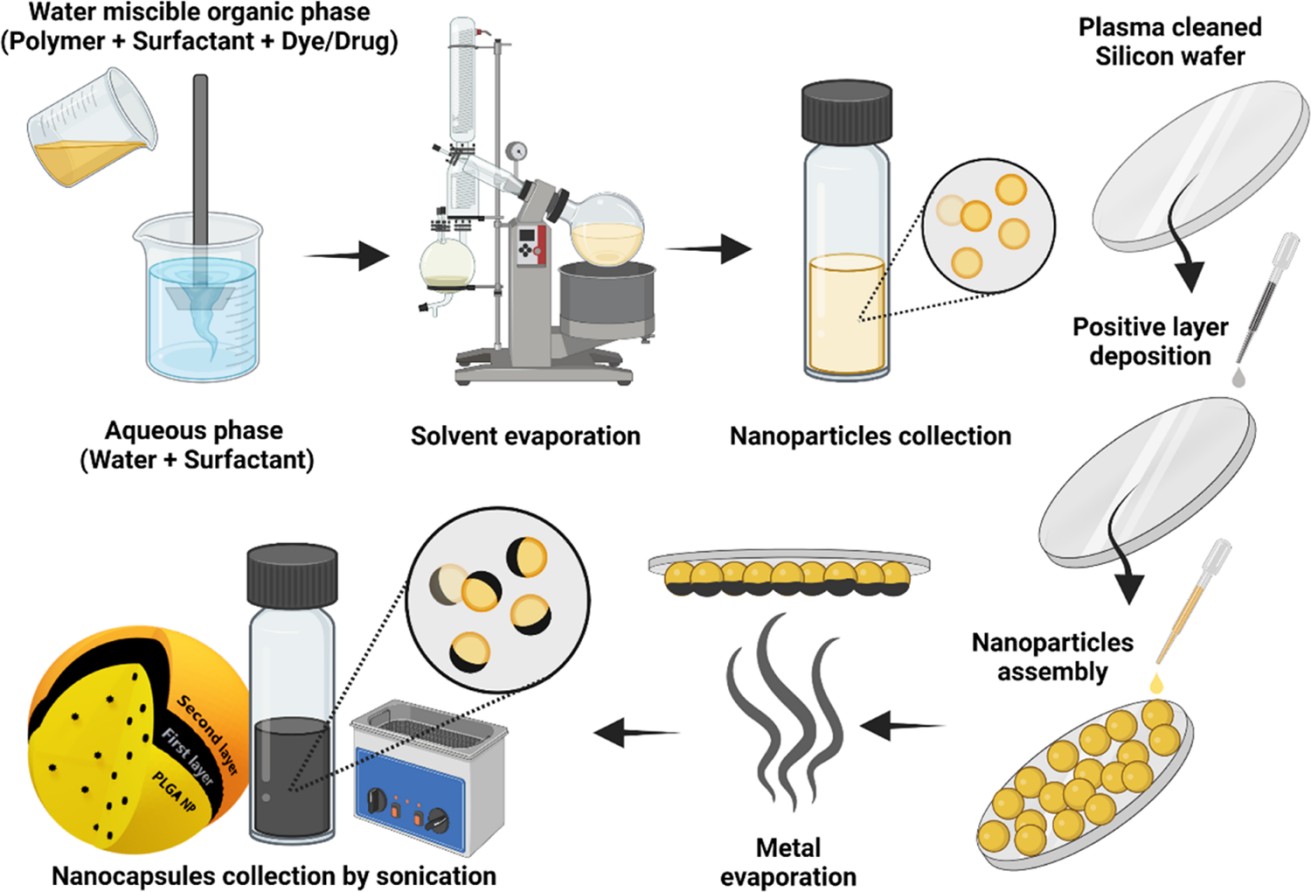
Schematic Representation of the Metal-Coated Janus Nanocapsule Fabrication The drug-loaded nanocapsules were prepared by the emulsification–evaporation technique, followed by their electrostatic self-assembly on positively charged silicon wafers to enable the deposition of the metal half-coating. The metal-coated nanocapsules were finally dispersed in water by bath sonication.

The metal-coated nanocapsule synthesis process is described in Scheme 1. Drug-loaded PLGA NPs were prepared using the emulsification–evaporation technique, as previously reported.^35^ Briefly, in the case of fluorescent nanocapsules, PLGA (100 mg), Tween 80 (0.01%), and coumarin-6 or rhodamine 6G (25 μg) were dissolved in 5 mL of acetonitrile. The other loaded nanocapsules were prepared similarly by substituting the coumarin with 0.2 mg/mL of paclitaxel or ICG in the organic phase. To generate the solid nanocapsules, the organic solution was gradually poured into 10 mL of an aqueous solution containing solutol (0.1%) while stirring for 15 min. Acetonitrile was evaporated using an evaporator (Basis Hei-Vap Value, Heidolph instruments, Germany) and the nanocapsules were collected by centrifugation and redispersed in double distilled water (DDW). In the particular case of erythromycin encapsulation (1 mg/mL), acetonitrile was replaced by ethyl acetate (3 mL), the amount of PLGA was increased to 120 mg, the solutol was replaced by 2% PVA, and the emulsion was generated by a 10 min probe sonication (Sonic ruptor 400, OMNI International, GA) after the stirring step.^36^

To self-assemble the loaded nanocapsules, silicon wafers (100 mm boron doped p-type Si wafer, University Wafers, MA) were cleaned and activated by oxygen plasma for 2 min at 100% power (Femto-QLS, Diener Electronics, Germany). Then, the wafers were immediately incubated with a 2% positive polyelectrolyte polymer PDDA in water for 5 min, rinsed with DDW, and dried under N_2_. The positively charged surface was incubated with the nanocapsule solution for 5 min and then rinsed and dried again to yield a self-assembled monolayer of well-separated nanocapsules on the Si surface.

The different metal films were deposited on the coated wafers using a high vacuum thin film electron beam deposition system (TFDS-462B, VST Ltd., Israel) inside a glovebox. The substrate holder temperature was set to −5 °C without rotation.

Detaching the nanocapsules from the silicon wafer surface is another critical step for obtaining concentrated dispersions of the metal–polymer hybrid NP solution. A sonication bath was used as intense mechanical vibration to properly detach the NPs. In our high-power ultrasonic bath (Sonorex Digiplus, Bandelin, Berlin, Germany), 5 min at 100% sonication power was adequate for removing the NPs from the wafers to a 0.2% PSS solution.

### 2.3 Morphology and Composition Characterization Using Scanning Electron Microscopy (SEM) and Transmission Electron Microscopy (TEM)/Energy-Dispersive X-ray Spectroscopy (EDS)

The morphology of the nanocapsules and their surface distribution on the wafer were examined using the environmental scanning electron microscope (SEM) Quanta 200 (FEI Company, The Netherlands) and the transmission electron microscope (TEM) Tecnai G2 F20 equipped with an energy-dispersive X-ray spectroscopy detector (EDS) (EDAX, TSL, AMETEK, Hillsboro, OR) for surface elemental identification. The chemical analysis was performed in the frame mode, representing the sum of the elemental composition of the imaged nanocapsules and the silicon substrate. In the case of Cu-PLGA samples, to avoid undesired signal from the equipment, electron energy loss spectroscopy (EELS) was performed for elemental mapping.

### 2.4 Particle Size, Charge, and Encapsulation Efficiency (EE%) Determinations

Particles were characterized according to their size, size distribution, and ζ potential. Dynamic light scattering (DLS) was performed at a scattering angle of 90° and at a temperature of 25 °C using a Zetasizer Nano ZSP (ZEN5600, Malvern Instruments, U.K.) to determine the mean diameter, polydispersity index (PDI), and electric charge of the nanocapsules. The nanocapsule morphology and size were examined and imaged using a transmission electron microscope (TEM) (JEM 1400Plus, JEOL, Japan, with a charge-coupled camera, Gatan Orius SC600). Samples were negatively stained with a 2% aqueous solution of uranyl acetate (1:1) and placed on a glow-discharged carbon-coated copper TEM grid (Ted Pella, Inc., Redding, CA). The mean size was calculated from at least 20 nanocapsules from two fields and presented as the mean ± standard deviation using ImageJ software. The encapsulation efficiency (EE%) was determined for each encapsulant thrice by centrifugation (10,000 rpm for 10 min) of 1 mL of the nanocapsule solution and resuspension in 1 mL of acetonitrile. The concentrations were measured by high-performance liquid chromatography (HPLC, Shimadzu, OR) using a C18 column for: (i) erythromycin (acetonitrile to 0.025 M ammonium dihydrogen phosphate in deionized water (60:40) at pH 7 and a detection wavelength of 205 nm)^37^ and (ii) paclitaxel (acetonitrile to DDW with 0.1% trifluoracetic acid (80:20) and a detection wavelength of 227 nm).^38^ The ICG concentration was evaluated using a SPECTRAFluor Plus plate reader (Tecan, San Jose, CA) in 780/820 nm Ex/Em. The encapsulation efficiency was calculated using the equation given below (eq 1)
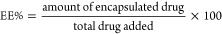
1

### 2.5 In Vitro Drug Release Profile

The loaded nanocapsules were removed from the wafers and counted, concentrated to 0.5 mL in deionized water by centrifugation, and then loaded into a dialysis tube (Pur-A-Lyzer Maxi Dialysis Kit MW cutoff 3.5 kDa, Sigma-Aldrich) that was immersed in dissolution media (8 mL, 1% Tween 80 in PBS) under constant stirring at 900 rpm. At fixed time intervals, 0.5 mL of the buffer solution was withdrawn and replaced with fresh buffer, and the amount of released fluorophore (coumarin-6) was evaluated using a plate reader in 440/528 nm Ex/Em.

### 2.6 Cell Lines

MDA-MB-231 breast adenocarcinoma tumor cells were purchased from ATCC (VA) and maintained in DMEM supplemented with 10% fetal calf serum (FCS) (Gipco, Brazil) in a medium with 1% penicillin/streptomycin (Biological Industries, Israel).

### 2.7 Magnet-Assisted Spheroid Formation

The magnetic attraction level of the Fe-PLGA nanocapsules was evaluated by using their capability to induce the aggregation of multicellular spheroids. For cell labeling, MDA-MB-231 cells were first plated at 7 × 10^3^ cells/well and incubated for 24 h; then, Fe-PLGA nanocapsules at a concentration of 1.4 × 10^9^ NCs/mL (equivalent to 5 μg/mL of iron) were added to the medium and incubated for 4 h to allow them to interact with the cancer cells. The spheroids were prepared as previously described, but with mild modifications.^39^ A preheated 2% agarose solution plated in 96-well plates at 50 μL/well and plate was incubated for 10 min for gelation. Cells were counted after trypsinization and seeded at 4 × 10^4^ cells/mL on top of the agarose-coated wells. A nickel-plated cylindrical neodymium magnet (magnetization N45, supermagnente, Gottmadingen, Germany), with a magnetic field and field gradient according to Figure S6b, was fixed under the desired wells, followed by overnight incubation for spheroid maturation. The spheroids’ brightfield and fluorescence images were taken with an ECLIPSE Ti2 inverted microscope (NIKON, Tokyo, Japan) to visualize the interaction of rhodamine 6G-loaded Fe-PLGA NCs with the spheroid and the manipulation of the spheroid inside the well using a magnet near the well wall. The viability of the spheroids was measured after 5 days of incubation with empty or paclitaxel-loaded Fe-PLGA nanocapsules by the WST-8 colorimetric assay (Sigma-Aldrich). The WST-8 reagent was added at a 1:10 ratio to the cell medium, and the plates were incubated for 3 h, after which absorbance was measured using a SPECTRAFluor Plus plate reader at 450 nm.

### 2.8 Photothermal Effects of Hybrid NPs

For the in vitro photothermal effect, Au-PLGA NPs were removed from a wafer and concentrated by centrifugation to 37 μg/mL of Au. The photothermal conversion efficiency at 808 nm was measured for: (i) commercial gold nanoshells (AuNS) and (ii) ICG-loaded and (iii) unloaded Au-PLGA NPs. The following equation (eq 2) was used^40^
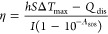
2where *h*, *S*, and *Q*_dis_ represent the heat transfer coefficient, area of the illuminated spot, and heat dissipated from the light absorbed by the polystyrene cell, respectively. In addition, *A*_808_ is the absorbance value of the nanocapsule dispersion at 808 nm, Δ*T*_max_ represents the temperature increase after 20 min of radiation, and *I* is the laser power (267 mW).

The visualization of ICG-loaded Au-PLGA NCs was performed by an *in vivo* imaging system (IVIS, PerkinElmer, Waltham, MA). For the analysis of the *in vivo* therapeutic effects, 8-week-old Hsd:Athymic Nude-Foxn1nu female mice were purchased (Harlan, Rehovot, Israel), stored in a specific pathogen-free (SPF) unit, and acclimated for a week. All animal procedures were conducted according to the institutional and national guidelines, and protocols were approved by the Hebrew University Ein Kerem Medical School IACUC (protocol MD-21-16572-5). Mice were injected subcutaneously (S.C.) with 5 × 10^6^ MDA-MB-231 cells/mouse in 100 μL PBS. Tumors were monitored until an approximate size of ∼120 mm^3^ was reached; then, the mice were divided into four groups. The loaded Au-PLGA NCs were injected intratumorally at a concentration of 1.82 × 10^11^ NCs/mL with a volume of 50 μL (i.e., 4 mg/kg of Au) into two of the groups, whereas the other two received only PBS intratumorally, and the mice were imaged by IVIS. Immediately after injection, the two groups were irradiated by a laser diode (B1-808-1500-15A, Sheaumann, MA, driven by a laser diode controller ITC4005, Thorlabs, Germany) with emission wavelength λ = 808 nm and light intensity of 1 W/cm^2^ for 5 min. The thermal images were acquired by an infrared thermal camera (8320P, Infrared Cameras Inc. (ICI), Beaumont, TX). The tumors were monitored, and their volumes were measured for 14 days post laser irradiation using the following equation (eq 3)
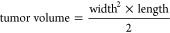
3Each tumor volume was normalized to its initial volume (*n* = 5). Five days post laser irradiation, the selected mice from the laser-treated groups were euthanized and the tumors were removed, sectioned, and stained with hematoxylin and eosin (H&E). Sections were blocked by 3% goat serum for 2 h, then stained with cleaved caspase-3 (1:500; cat. no. 9664, Cell Signaling Technology, Inc.) overnight at 4 °C, followed by 2 h of incubation with Cy5 antibody (Alexa Fluor 647, ab150083, abcam) and 20 min of counterstaining with 6-diamidino-2-phenylindole (Dapi), and then coverslipped and imaged by a fluorescence microscope.

### 2.9 Reactive Oxygen Species (ROS) Production

Gold-capped PLGA nanocapsules loaded with ICG were examined for their ability to generate reactive oxygen species, using 2′,7′-dichlorodihydrofluorescein diacetate (DCFH-DA) staining^41^ and its effect on cell viability. Cells were plated at 4 × 10^4^ cells/well 24 h prior to treatment for 1 h with 3 × 10^9^ nanocapsules/mL (i.e., 12 μg/mL) of ICG-loaded or unloaded Au-PLGA nanocapsules. Then, the wells were radiated using a laser diode (λ = 808 nm at 1 W/cm^2^) for 0, 2, 5, or 10 min, and the medium was replaced by 100 μL of medium containing 10 μM DCFH-DA. The plates were incubated for 1 h and washed three times, 100 μL of fresh medium was added, and fluorescence was read immediately using ex/em 485/530. The results were normalized by the fluorescence readout from untreated cells, thus being presented as the reactive oxygen species increase factor. The cell viability was evaluated by the MTT assay 72 h post laser radiation. Cells were incubated with 0.5 mg/mL of MTT reagent for 4 h. Then, the supernatant was removed and dimethyl sulfoxide (DMSO) was added. The absorbance was read at 570 nm by a SPECTRAFluor Plus plate reader.

### 2.10 Antibacterial Effect of Cu-PLGA NPs

Copper-capped PLGA NPs loaded with the antibiotic erythromycin were evaluated for their effect on Gram-positive *Staphylococcus aureus* (*S. aureus* C127). Bacteria (6 × 10^5^ CFU/mL in lysogeny broth) were incubated with erythromycin (0.02–12 μg/mL) in 96-well plates at 37 °C. The minimum inhibitory concentration (MIC) was determined by measuring the optical density (OD), at 600 nm, for each well every 20 min over a period of 20 h using the Eon microplate spectrophotometer (Gen5 v2.07, BioTek Instruments, VT).^42^ To compare between treatments, the lowest concentration of the antibiotic to inhibit 50% of the isolates (MIC50) was measured. Uncoated erythromycin-loaded PLGA NPs were tested for their ability to inhibit bacterial growth using an erythromycin concentration range from 0.02 to 12 μg/mL, which was determined by HPLC. Erythromycin-loaded Cu-PLGA NCs at concentrations with an erythromycin concentration from 9.6 × 10^–3^ to 0.6 μg/mL were studied using the same method to evaluate the synergistic effect of copper in the delivery system by subtracting the background absorbance from the NCs. To examine the morphological changes, TEM images of the bacteria were acquired after 2 h of treatment. The colony-forming units (CFU/mL) of each treatment were measured after 20 h of treatment by decimally diluting the sample 8 times, and the colonies were counted after 24 h of incubation in LB-agar plates spotted with 10 μL from each dilution at 37 °C.

### 2.11 Statistical Analysis

All data were analyzed with GraphPad Prism 8.0.1 software. Results are presented as mean ± standard error. One-way analysis of variance (ANOVA) was applied in the statistical difference comparation. In the cases in which the data did not follow a normal distribution, specifically in the context of the Au-PLGA in vivo experiment, statistical analysis was performed using a Kruskal–Wallis test to assess differences among multiple groups. Subsequently, a Dunn’s post hoc test was conducted to compare pairs of groups to evaluate the therapeutic effect of the treatment. Statistically significant differences were determined as *p < 0.05, **p < 0.01, and ***p < 0.001.

## 3 Results and Discussion

### 3.1 Drug-Loaded Nanocapsule Fabrication and Metal Coating

The drug-loaded PLGA nanocapsules were fabricated using an established method of emulsification–evaporation according to the details described in Section 2.^35^ Highly uniformed nanocapsules with diameter ca. 100 nm were obtained, with a PDI lower than 0.1 (Table 1) and negative charge (ζ potential −30.2 mV). Prior to metal deposition, we developed a robust protocol for the electrostatic self-assembly of the PLGA nanocapsules on silicon surfaces to enable metal evaporation on only half of their surface. The silicon wafers were first activated by plasma, resulting in the formation of silanol groups (−Si–OH)^43^ and then coated with a monolayer of the positively charged polyelectrolyte PDDA, which facilitated the electrostatic attachment of the negatively charged PLGA nanocapsules to the surface. When examining the nanocapsule distribution and morphology on the wafer surface by SEM, it was found that PDDA was the most adequate linker, providing dense and well-separated nanocapsules on the surface in comparison with the other tested positive molecules (PAH and APTMS) (Figure S1).

**Table 1:** Physicochemical Characteristics of NPs before and after Metal Depositions

nanocapsule	layers	mean size by DLS (nm)	PDI	mean size by TEM ± SD (nm)	ζ potential (mV)	encapsulant	encapsulation efficiency ± SD (%)
PLGA		108.1	0.07	106 ± 11	–30 ± 3		
Fe-PLGA	Fe—20 nm	138.8	0.16	124 ± 12	–19 ± 2	paclitaxel	50 ± 3
	SiO_2_—10 nm						
Au-PLGA	Ti—2 nm	131.2	0.31	117 ± 11	–34 ± 1	indocyanine green	55 ± 3
	Au—20 nm						
Cu-PLGA	Cu—20 nm	191.7	0.31	149 ± 32	–36 ± 1	erythromycin	36 ± 3
	SiO_2_—10 nm						

To form the metal (iron, gold, or copper) semishell that facilitate efficient release of the loaded drug, we used highly directional electron beam evaporation on the self-assembled arrays of the loaded PLGA nanocapsules. Since iron (Fe) and copper (Cu) tend to oxidize over time, an additional 10 nm silica (SiO_2_) layer was deposited over the 20 nm metal layer as a protective shield.^44^ To guarantee the firm attachment of the 20 nm Au layer to the PLGA nanocapsules and wafer, a 2 nm Ti layer was first deposited. Note that the direct deposition approach is a straightforward method to control the thickness of the metal layers, which allows fine-tuning their functional properties. For example, by changing the thickness of the shell layers, it is possible to control the magnetization reversal in the Fe coating or to tune the wavelength of the plasmonic resonance of Au and the photothermal response.^45^ It is also important to emphasize that the growth of these anisotropic multilayers on the polymer particles (e.g., SiO_2_ on Fe or Cu as protection from oxidation) to optimize their performance would be extremely complex by using chemical synthesis methods. Notably, although in our case we have used the additional layers for structural purposes, the same concept could be used to introduce new functionalities in the structures. For example, combining Fe and Au layers can provide dual magnetic resonance and X-ray contrast for noninvasive imaging, Fe and platinum (Pt) could merge magnetic imaging with antineoplastic capabilities, and Cu and silver (Ag) could merge the antibacterial capabilities and enable efficient photothermal actuation to boost the therapeutic effects even further.^46^

**Figure 1:**
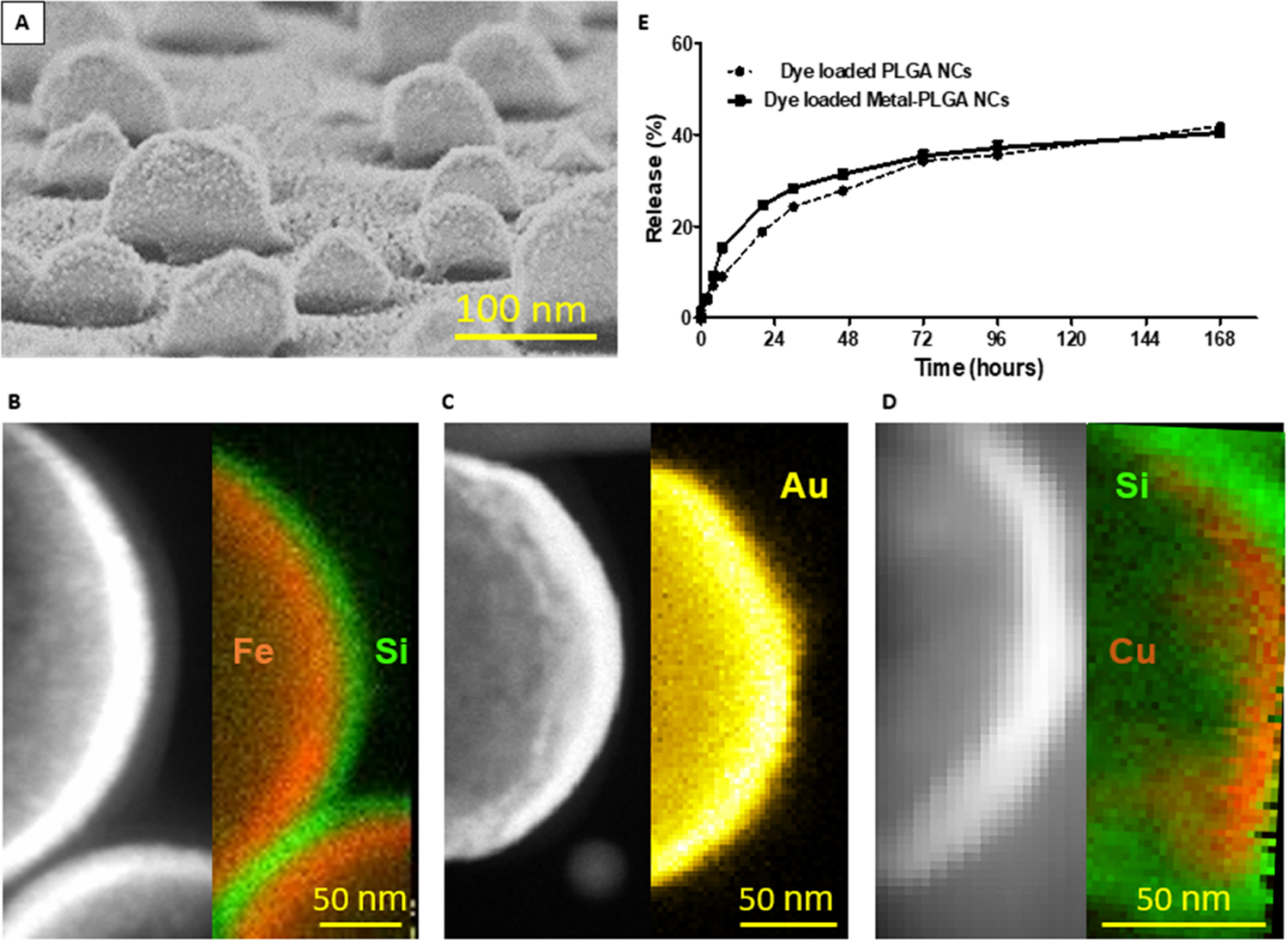
(A) SEM image of metal-coated nanocapsules on the wafer. TEM images and element mapping of representative metal-coated nanocapsules corresponding to (B) Fe (red) and Si (green) in the Fe-PLGA nanocapsules, (C) Au (yellow) in the Au-PLGA nanocapsules, and (D) Cu (orange) and Si (green) in the Cu-PLGA nanocapsules. (E) Release profile of the dye loaded into uncapped and metal-capped PLGA NPs over time.

The SEM images of Figure 1A show the expected array of self-assembled nanocapsules coated with the metal layer on their upper half, whereas the TEM images with EDS/EELS mapping analysis verify the presence and thickness of the different deposited layer, i.e, Fe/SiO_2_ for the Fe-PLGA nanocapsules (Figure 1B), Ti/Au for the Au-PLGA nanocapsules (Figure 1C), and Cu/SiO_2_ for the Cu-PLGA nanocapsules (Figure 1D). Both the self-assembly of the loaded PLGA nanocapsules and the final dispersion of the metal-capped nanocapsules were optimized to maximize the final concentration and to minimize the generation of particle aggregates, yielding a ca. 85% recovery from the wafer (Figures S2 and S3D,E).

To compare the drug release profiles and kinetics of the PLGA nanocapsules with and without metal deposition, release studies were performed by detecting the druglike fluorophore molecule coumarin-6. Interestingly, no significant differences were observed in the release profile and kinetics between the metal-coated and uncoated nanocapsules (∼41% over 168 h), being similar to other reports on the release kinetics of coumarin-6 from PLGA NPs (33–45% over 168 h).^47−49^ A similar release pattern of the paclitaxel drug was also observed in our previous work for Fe-coated and uncoated polymer nanoparticles.^33^ These results indicate that neither the metal layer nor the deposition process reduced the percentage and extension of the drug release (Figure 1E), thereby preserving the water hydrolysis of the polymer chains that triggers the polymer degradation.^50^ In the case of the ICG-loaded nanocapsules, a similar release pattern is expected, considering that the formulation of the polymer nanocapsules loaded is identical to that in the paclitaxel or coumarin cases, which is confirmed with the observed therapeutic activity of the drug.

### 3.2 Physicochemical Characterization

The main physicochemical parameters of the uncoated and coated nanocapsules are summarized in Table 1. The DLS measurements showed slightly larger nanoparticle diameters with respect to the TEM measurements (Figure S4), as expected from their larger hydrodynamic diameter. In the case of the Cu-coated nanocapsules, the difference is larger due to the presence of small aggregates in the self-assembled monolayer, resulting from the different formulations of the PLGA nanocapsules. In all of the examined cases, a stable colloidal dispersion was obtained with negative ζ potential values ranging from ∼−18 to −36 mV. The high polydispersity index (PDI) values observed in Au- and Cu-coated nanocapsules are due to their high optical anisotropy, which generates different scattering intensities depending on the particle orientation with respect to the polarization of the incident light, thus overestimating the actual polydispersity observed in the SEM images. In the case of Fe-coated nanoparticles, the PDI is lower as a result of the highly damped plasmonic behavior of the nanostructured Fe,^51,52^ which reduces the optical anisotropy. Therefore, for optically anisotropic structures, SEM/TEM or other nanoparticle analysis techniques should be used to accurately determine the PDI.^53,54^ Finally, the nanocapsules exhibited a loading efficiency over 50% for paclitaxel and ICG, whereas it was slightly lower (36%) for erythromycin. The typical optical absorbance values of the dispersed Fe-, Au-, and Cu-coated nanocapsules are gathered in Figure S5, showing the expended broadband absorption for the Fe-PLGA NCs and more defined plasmonic bands for the Au- and Cu-coated particles, although the Cu-PLGA NC absorbance was significantly lower.

Next, we analyze the synergistic effects that can be achieved for the different metal-PLGA-drug formulations.

### 3.3 Fe-PLGA Nanocapsules Loaded with Paclitaxel for Magnetically Enhanced and Manipulated Cell Spheroids with Boosted Therapeutic Effect

The Fe-coated nanocapsules offer a very efficient external magnetic manipulation, which is granted by their large volume, V, and the strong ferromagnetism of the metal Fe layer, whose saturation magnetization, M_S_, is 3-fold higher than that of the typical iron oxides used for biomedical applications. This confers them a total moment, *m* = *V* × *M*_S_, ca. 250 times larger than a Fe_3_O_4_ nanoparticle of 20 nm. The diameter and thickness of the Fe layer have been designed to exhibit a magnetic vortex (Figure S6a),^33,45^ thereby showing near zero remanence and enabling high colloidal stability, despite the large content of ferromagnetic material. This is due to the negligible magnetic dipole–dipole interactions between the coated nanocapsules in the absence of an external magnetic field. However, in the presence of a magnetic field gradient, the large induced magnetic moment in the Fe semishell allows the generation of magnetophoretic forces that are 2 orders of magnitude larger than those in superparamagnetic iron oxide nanoparticles.^33^ Here we exploit the high drug-loading capacity and the magnetic strength to demonstrate the ability to (i) magnetically manipulate cells and improve the efficiency in the formation of three-dimensional (3D) cell cancer spheroids and (ii) enhance the effects of encapsulated compounds. While some previous studies have demonstrated magnetically induced multicellular spheroids using NPs that drive cell aggregation,^55^ in our case, the Fe-PLGA nanocapsules enable a simultaneous combination of magnetic actuation and drug release in the same system, thus accelerating cell assembly at low particle concentrations.

**Figure 2:**
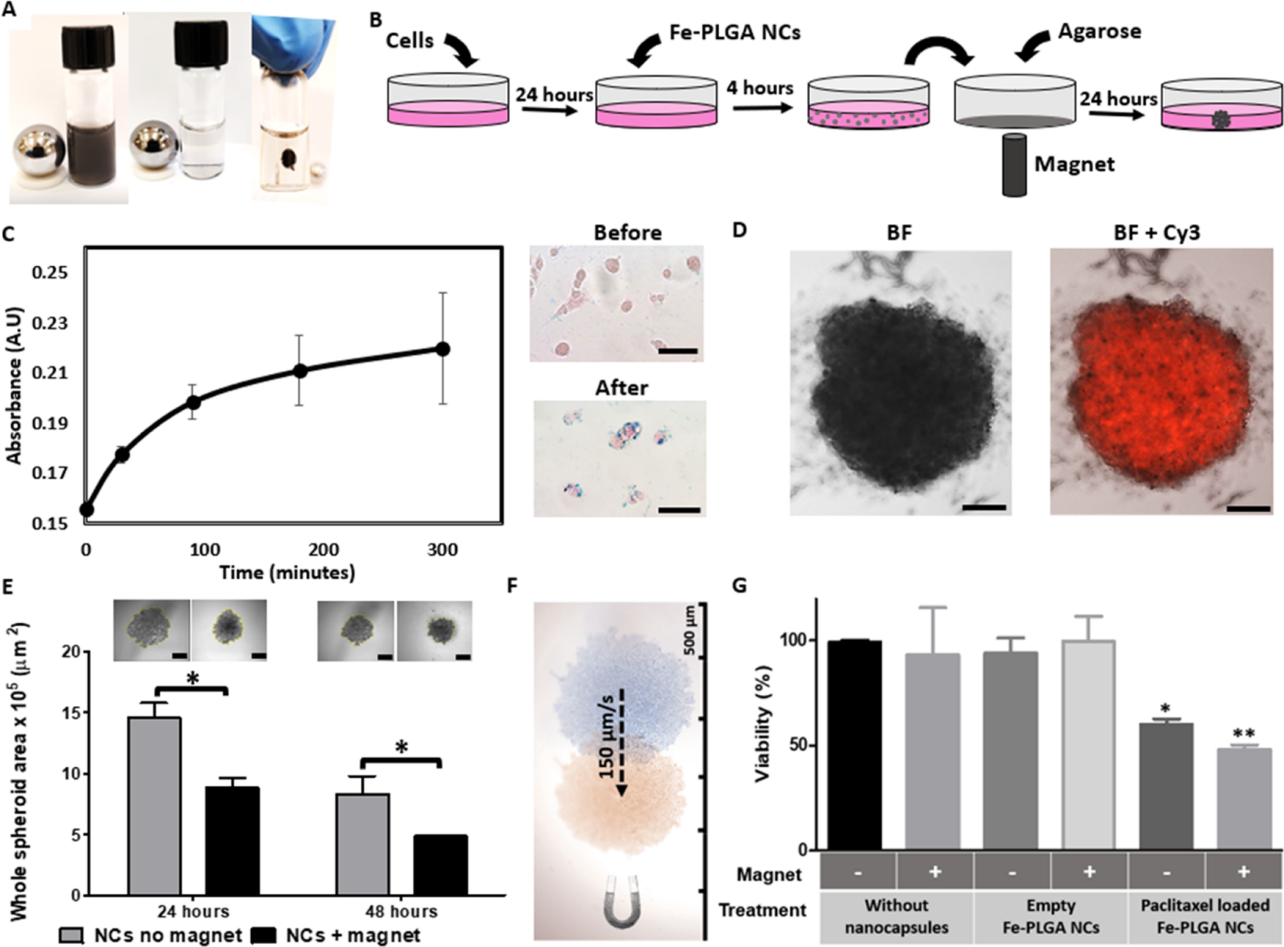
Fe-PLGA nanocapsules for the magnetic manipulation of spheroids. (A) Picture showing the magnetic concentration of dispersed nanocapsules after 90 s. (B) Schematic illustration of the magnetically assisted spheroid formation. (C) Absorbance at 590 nm of MDA-MB-231 cells incubated with Fe-PLGA nanocapsules for 5 h and the eosin/Prussian blue staining of cells before and after incubation (scale 50 μm). (D) Fluorescence and brightfield (BF) images of spheroids containing rhodamine 6G-loaded Fe-PLGA nanocapsules after 48 h (scale 200 μm). (E) Brightfield microscopy images (scale 500 μm) and cross-section area measurements of the spheroids, formed with empty Fe-PLGA nanocapsules with and without magnetic concentration after 24 and 48 h. (F) Spheroid magnetic manipulation inside the well showing the positions before (blue) and after (orange) using an external magnetic field gradient. (G) Viability of the spheroids formed without nanocapsules, with empty Fe-PLGA nanocapsules and paclitaxel-loaded Fe-PLGA nanocapsules with (+) or without (−) magnetic manipulation.

The high magnetic strength of the Fe-PLGA nanocapsules is reflected in Figure 2A, in which the response to an external magnetic force was demonstrated by placing a magnet close to a concentrated solution of Fe-PLGA nanocapsules, resulting in complete attraction and accumulation of all of the nanocapsules to the magnet in 90 s.

To enable cell actuation with magnetic forces for improving the assembly of the spheroids, the nanocapsules should be efficiency-internalized or adhered at the cell surface before placing the magnet to trigger the cell concentration (as illustrated in Figure 2B). MDA-MB-231 cancer cells were analyzed for their uptake capacity during 5 h of incubation with the Fe-PLGA nanocapsules at a concentration of 1.4 × 10^9^ nanocapsules/mL (i.e., an Fe concentration of 5 μg/mL). After intensive washes with PBS, the nanocapsules’ uptake was assessed by measuring the absorbance at 590 nm (Figure 2C; left panel), which corresponds to the absorbance peak of the nanocapsules (Figure S4). After an initial increase of the absorbance signal in the first 2 h, no major difference occurred between 3 and 5 h. As a result, we selected 4 h as the optimized uptake time. The confocal fluorescence images of cells incubated with the Fe-PLGA nanocapsules loaded with rhodamine 6G for 4 h showed a large quantity of internalized nanocapsules inside the cells, although a significant fraction of particles was still at the cell membrane (Figure S7). In contrast, after 24 h of incubation, the majority of the nanocapsules was already internalized (Figure S7). Prussian blue staining to visualize the presence of iron showed an intense signal in the treated cells after 4 h of incubation compared to the nontreated cells (Figure 2C; right panel).

To evaluate both the cellular uptake and the distribution of the nanocapsules in the spheroids, Fe-PLGA nanocapsules loaded with rhodamine 6G showed a qualitatively high uptake of the nanocapsules with a very homogeneous distribution in the spheroid after 24 h of assembly (Figure 2D).

To compare the spheroid formation rate with and without magnetic actuation, the cross-section area of the formed spheroid was calculated from images taken at 24 and 48 h, showing a significant decrease (40%) in the spheroid area for the magnetically actuated cells, thus indicating the formation of more condensed and stable spheroids (Figure 2E).^55^ Such efficient magnetic actuation is not only important for improving spheroidal formation but can also be useful for externally manipulating the whole spheroid in an aqueous medium, as can be observed in Figure 2F and the video in the Supporting Information. These images showed the capability to magnetically displace the spheroids inside the well at a speed of 150 μm/s, despite the low nanocapsule concentration.

To validate the synergistic effect of an active compound loaded in the nanocapsules, we used paclitaxel-loaded Fe-PLGA NCs to evaluate the anticancer therapeutic effect in breast cancer carcinoma spheroids. Whereas spheroids treated with empty Fe-PLGA nanocapsules or PBS were still vital after 5 days, paclitaxel-loaded Fe-PLGA NPs decreased the viability to 60 and 48% in the unmanipulated and magnetically manipulated spheroids, respectively (Figure 2G), thus highlighting the magnetically enhanced therapeutic effect. This is probably due to the higher drug concentration in the magnetically actuated spheroids resulting from their higher cell (and nanocapsule) density.

This magnetically controlled spheroid model may be relevant for the screening of drug candidates in more physiologically relevant cancer models.^56^ In addition, there is a major limitation regarding the penetration of drug carriers into tissues and 3D cultures, such as spheroids. Therefore, locating the drug in the center of the cell mass, as shown in our study, may potentially facilitate the assessment of the drug responses in spheroids or other 3D cultures.^57^ This could be utilized in personalized medicine for screening anticancer drugs on spheroids obtained from patient’s biopsies.^57^

The magnetic nanocapsules could also be applied for tissue engineering, as spheroids could be magnetically assembled in complex shapes and scaffolds.^58^ Moreover, the encapsulation of growth factors inside Fe-PLGA nanocapsules could improve the assembly of the spheroids and support long-term culture, as previously shown with PLGA microspheres loaded with transforming growth factor-β1 (TGF-β1) in human mesenchymal stem cell spheroids.^59^ The concentration of the Fe-PLGA nanocapsules used for magnet-assisted spheroid formation (5 μg/mL) is in line with concentrations used with the commercially available NanoShuttle-PL superparamagnetic iron oxide nanoparticles (SPIONs),^55^ but without the drug-loading capability. It is also worth mentioning that the metallic iron semishell in the nanocapsules can also provide a high photothermal conversion efficiency and a high contrast in magnetic resonance imaging,^33^ thereby expanding the repertoire of functionalities of these nanocapsules for therapeutic applications.

### 3.4 Synergistic Photodynamic/Photothermal Therapy and Noninvasive In Vivo Imaging Using Indocyanine Green (ICG)-Loaded Au-PLGA Nanocapsules for Tumor Eradication

**Figure 3:**
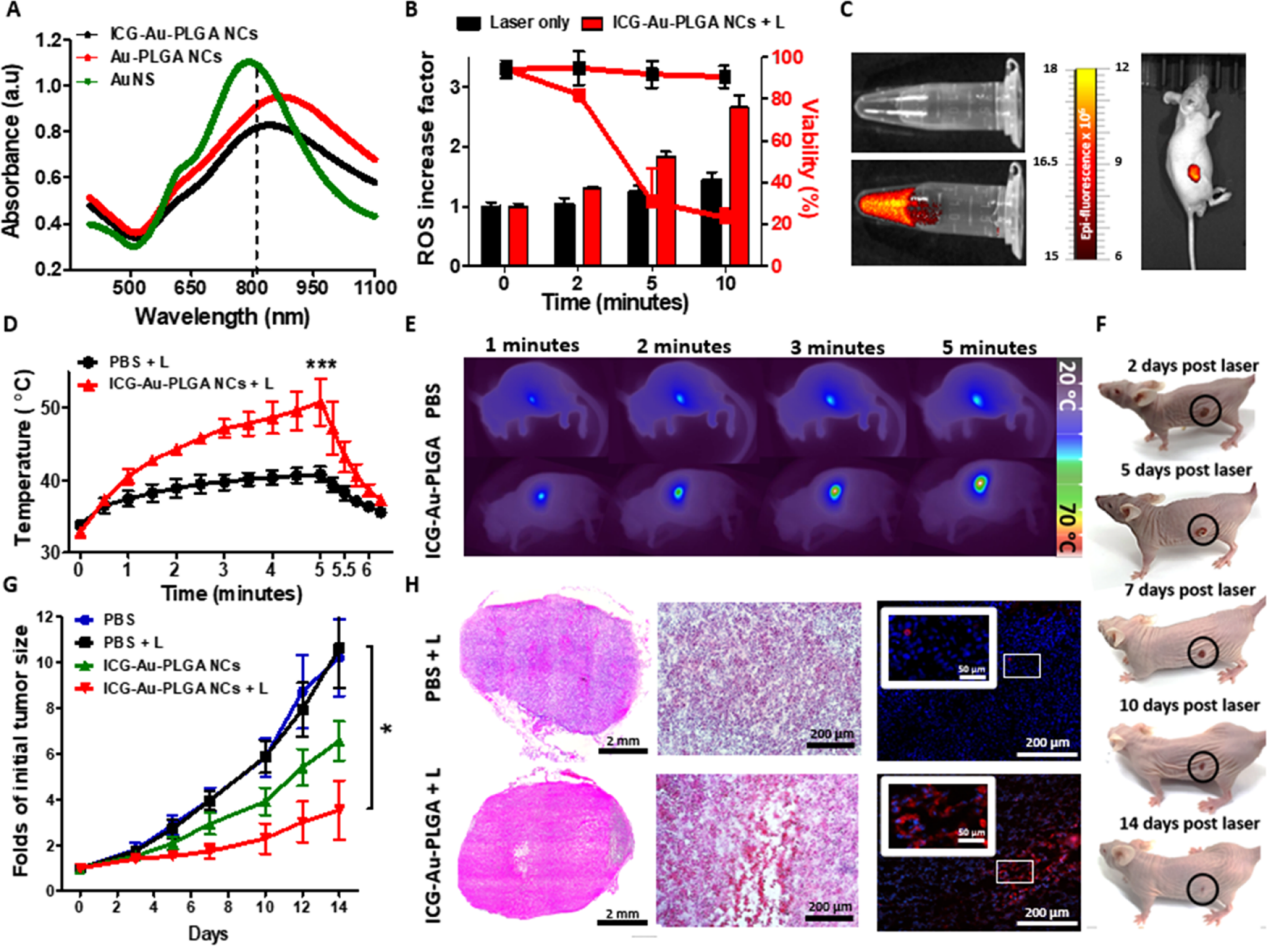
Application of Au-PLGA nanocapsules as dual photodynamic and photothermal therapy. (A) Visible–NIR spectrum of Au-PLGA nanocapsules and nanoshells. The vertical line indicates the wavelength used in the experiments, λ = 808 nm. (B) ROS production (bars) and viability (lines) of untreated and ICG-loaded Au-PLGA NC-treated MDA-MB-231 cells vs irradiation time (808 nm, 0.95 W/cm^2^). (C) Fluorescence images of ICG-loaded Au-PLGA NCs in solution compared to PBS and after the intratumoral injection. (D) Photo-induced temperature increase by ICG-loaded Au-PLGA nanocapsules in vivo in MDA-MB-231 tumor-bearing mice (808 nm, 1 W/cm^2^), and (E) representative thermal camera images. (F) Photographs of the mouse treated with Au-PLGA nanocapsules and laser at different time points during the experiment. (G) Evolution of the tumor volumes post radiation. (H) H&E and caspase-3 staining of the mice sacrificed on day 5 treated with laser and PBS or ICG-loaded Au-PLGA NCs (blue represents Dapi and red represents cleaved caspase-3; scale: 200 μm and insert scale: 50 μm).

Gold NPs have been widely utilized to generate photothermal therapies with near-infrared (NIR) radiation to locally induce cancer cell death.^60^ Conversely, the small molecule ICG has been used as a photodynamic agent due to its capability to generate reactive oxygen species (ROS) upon NIR irradiation.^61^ Therefore, simultaneous photothermal and photodynamic local actuation could enhance the nanotherapy performance by combining (i) the oxidative stress produced by the photodynamic agent and (ii) the hyperthermia irreversible cell damage. To demonstrate the capacity to merge both effects, we fabricated Au-PLGA nanocapsules loaded with ICG following the same fabrication strategy. The Au thickness and size of the PLGA particles were selected to exhibit strong and broadband plasmonic resonance within the first biological window (peak at 870 nm) (Figure 3A) to ensure high light penetration in the tissues. The incorporation of ICG in the capsules induces a slight blue shift of the resonance (Figure 3A), as a consequence of the ICG absorption band centered at approximately 780 nm.

Next, we analyzed the photothermal conversion efficiency at a typical wavelength used in the first biological window (i.e., 808 nm) for unloaded and ICG-loaded Au-PLGA nanocapsules, compared to commercial Au nanoshells over silica nanoparticles that have been widely used in photothermal therapies.^62^ As Table 2 shows, the photothermal efficiencies present remarkably high 76 and 79% values for the unloaded and ICG-loaded Au-PLGA nanocapsules, respectively (Figure S8). Therefore, the incorporation of ICG does not affect the photothermal efficiency. In contrast, standard gold nanoshells with 20 nm Au and a resonance at 800 nm showed an efficiency of only 50%. Such photothermal efficiency enhancement is due to the nanodome shape of the Au coating in the nanocapsules, which enables a drastic reduction of the scattering cross section compared to complete nanoshells.^45^

**Table 2:** Photothermal Conversion Efficiency Comparison

sample	Abs at 808 nm	Δ*T* (°C)	η
AuNS	1.09	10.5	0.50
Au-PLGA NCs	0.91	14.8	0.76
ICG-Au-PLGA NCs	0.82	14.5	0.79

To explore the synergistic photothermal and photodynamic effects, ICG-loaded Au-PLGA nanocapsules (1.5 × 10^10^ NCs/mL, equivalent to 60 μg/mL of Au) were added to 4 × 10^4^ cell/mL MDA-MB-231 cells and evaluated for ROS production after laser irradiation (808 nm, 0.95 W/cm^2^). Compared to untreated cells, the cells treated with ICG-loaded Au-PLGA nanocapsules produced a significantly higher ROS concentration as the exposure time increased, being about 2-fold higher after 10 min of irradiation. These ROS levels directly correlated with the drastic decrease in cell viability compared to nontreated cells after 5 min of irradiation (Figure 3B).

The high fluorescence of the ICG-loaded nanocapsules was demonstrated by the IVIS device (Figure 3C; left panel), thus showing that there is no significant fluorescence quenching by the Au semishell. Importantly, the ICG-loaded nanocapsules can be used for noninvasive visualization in vivo with very strong contrast (Figure 3C; right panel).

To study the efficacy of the photothermal–photodynamic nanotherapy in vivo, MDA-MB-231 tumor-bearing mice were treated with intratumoral injection (50 μL) of either PBS or ICG-loaded Au-PLGA nanocapsules (1.82 × 10^11^ NCs/mL equivalent to 4 mg of Au per kg of mouse). The mice were imaged by IVIS to visualize the presence of ICG inside the tumor (Figure 3C), showing a homogeneous distribution.

Next, a 5 min laser exposure was monitored via thermal imaging of the tumor site, which increased the local temperature by 6.9 °C for the PBS-treated group and 16.6 °C for the nanocapsule-treated group (Figure 3D,E). The healing from the radiation was monitored for 2 weeks post injection, demonstrating almost complete healing of the radiated area (Figure 3F). A substantial reduction in tumor growth was exhibited with the nanocapsule- and laser-treated group (3.5-fold of the initial tumor volume), compared to the groups of nanocapsules without laser, only laser, and PBS control (6.5, 9.3, and 9.1, respectively; Figure 3G). The sectioned tumors, stained by H&E and cleaved caspase-3 to evaluate necrosis and apoptosis, displayed a necrotic core and higher levels of apoptotic fluorescence signals for the laser- and nanocapsule-treated mice, compared with the laser-treated mice injected with PBS, as a consequence of the combination of the higher photothermal effect and the oxidative stress caused by ICG^61^ (Figure 3H). Our observations supported the notion that the combination of photodynamic and photothermal therapies using ICG and gold-containing formulations can lead to efficient anticancer effects in vivo.^63−65^ In contrast to these technologies in which ICG is either conjugated to gold nanoclusters,^63^ acting as a coating layer on the gold cluster cores,^64^ or coencapsulated with gold nanorods in a polymer matrix,^65^ our approach enables more versatile combinations and adjustments of the gold layer thickness and potentially supports the coencapsulation of ICG with additional drugs inside the NCs.

In addition to the photothermal effects, the Au semishell also provides an intense X-ray contrast,^45^ which can be useful for noninvasive deep tissue imaging by computed tomography. It is also possible to integrate Fe and Au layers that can complement the optical treatment with magnetic guidance to enhance the therapeutic effects locally. The Fe/Au combination could also enable simultaneous opto-magnetic heating thermometry in vivo.^31^

### 3.5 Synergistic Antibacterial Treatment with Cu-PLGA Nanocapsules Loaded with Erythromycin

**Figure 4:**
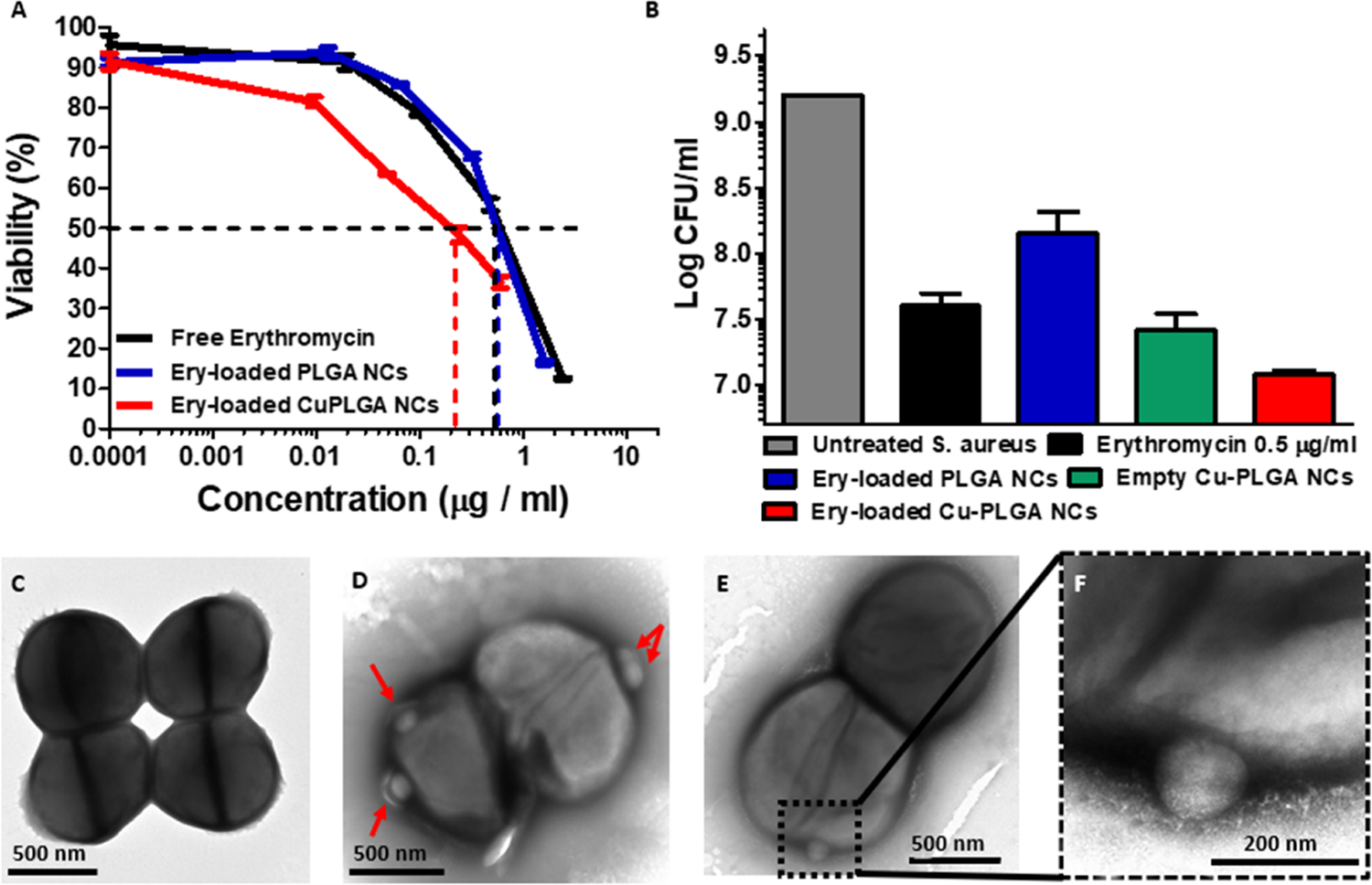
Synergistic antibacterial activity of Cu-PLGA nanocapsules. (A) Antibacterial activity of free erythromycin, PLGA-erythromycin, and Cu-PLGA-erythromycin NCs against *S. aureus* as a function of the erythromycin concentration, evaluated by the optical density method after 20 h of incubation. (B) Determination of the colony-forming units (CFU per mL) after 20 h treatment of free erythromycin (0.5 μg/mL) compared to loaded PLGA nanocapsules, unloaded Cu-PLGA nanocapsules, and loaded Cu-PLGA nanocapsules at a concentration of 2.5 × 10^10^ nanocapsules/mL. TEM images of (C) untreated and (D–F) erythromycin-loaded Cu-PLGA nanocapsule-treated bacteria (magnification 10k and 40k).

Copper, among other metals, has been widely studied for its antibacterial activity due to its ability to inhibit the growth of a wide spectrum of microorganisms.^66^ The antibacterial mechanism of the action of copper is based on “contact killing” and was linked to the capacity of the metal to release copper ions, which causes membrane damage and eventually DNA degradation.^67^ A combination of a commercial antibiotic drug with this metal may potentially enhance the antimicrobial activity. Therefore, erythromycin was loaded in Cu-PLGA nanocapsules to evaluate the antibacterial activity against the Gram-positive bacteria, *S. aureus*. To determine the minimum inhibitory concentration, MIC50 *S. aureus* cultures were first treated with free erythromycin at concentrations of 0.02–12 μg/mL. The MIC50 value was about 0.5 μg/mL (Figure 4A). The antibacterial activity of the uncoated erythromycin-loaded PLGA nanocapsules was tested on *S. aureus* to compare the activities of the free and loaded drugs, showing a similar inhibition profile after 20 h of incubation (Figure 4A), with a MIC50 erythromycin value of 0.5 μg/mL. Erythromycin concentrations were determined by HPLC (Figure S9). To evaluate the synergistic bactericidal effect, MIC50 was also measured in erythromycin-loaded Cu-PLGA nanocapsules, showing a remarkable 2.5-fold lower MIC50 concentration value (i.e., 0.2 μg/mL, equal to 2.5 × 10^10^ NC/mL) (Figure 4A), thus indicating the powerful concomitant effect of the Cu cap. After 24 h of incubation at 37 °C, copper ion release from Cu-PLGA NCs was observed, with approximately 80% of the initial copper content released to the medium (Figure S10).

In addition, the antibacterial effects of copper and the antibiotic drug were evaluated by determining the colony-forming units (CFU/mL) after 20 h of different treatments using the MIC50 concentration. As can be observed in Figure 4B, the free erythromycin at 0.5 μg/mL resulted in 4 × 10^7^ CFU/mL, i.e., significantly lower than untreated bacteria (1.6 × 10^9^ CFU/mL). To highlight the effect of Cu, we also analyzed the CFU for the unloaded Cu-PLGA NCs and erythromycin-loaded Cu-PLGA nanocapsules in comparison to uncapped erythromycin-loaded PLGA nanocapsules at the same particle concentration of 2.5 × 10^10^ NC/mL, achieving a remarkable reduction up to 2.6 × 10^7^ and 1.42 × 10^8^ CFU/mL compared to 1.2 × 10^7^ CFU/mL, respectively. These results demonstrate the synergistic effects of the Cu layer and the antibiotic drug.

To clarify how the erythromycin-loaded Cu-PLGA nanocapsules affect the bacteria, we used TEM to examine the morphological changes that occur in *S. aureus* after 2 h of treatment. Untreated bacteria displayed a normal undamaged structure (Figure 4C). In contrast, erythromycin-loaded Cu-PLGA nanocapsules interacted with the outer layer of *S. aureus*, resulting in an amorphous shape, with blurry and disturbed membranes (Figure 4D–F, nanocapsules marked in red). The bright areas in the center also indicate a decrease in cytoplasmatic material due to the membrane damage.^68,69^

In addition to Cu, decorations with other metals that are known for their bactericidal activities such as silver, zinc, titanium, nickel, and palladium could be used to enhance the antibacterial effects.^70^ These types of metal-coated nanocapsules containing antibiotic, antifungal, or antiviral drugs circumvent the necessity of using conjugating, stabilizing, and reducing agents during the fabrication of metallic nanoparticles loaded with antimicrobial drugs.^71^ Moreover, since one of the greatest risks to human health is the increasing antibiotic resistance, the reactive metals can be an interesting alternative to eradicate resistant bacteria, allowing the administration of lower initial doses.^72^ Our results are in line with other studies on metal–antibiotic combination effects on *S. aureus*, specifically with the macrolide family, where erythromycin (10 μg disks) dipped in silver NP solution increased the zone of inhibition by 68% compared to undipped disks,^73^ and the azithromycin (15 μg disks) zone of inhibition was increased by 48 and 11% when the disks were impregnated with silver NP or zinc NP solution, respectively.^74^

Furthermore, the combination of Cu and Fe layers could also offer additional functionalities to locally enhance the therapeutic effects by magnetic manipulation and to boost even further the therapeutic effects by photothermal actuation.

## 4 Conclusions

Transitioning from single-function nanocarriers to multifunctional ones, which integrate multiple components in a single system, has been recognized as an important modality for therapy and theranostics.^12^ This is in line with the growing acknowledgment that a single mechanism of action is typically insufficient to treat complex diseases featuring multiple and parallel biological processes.^75^ In this study, we established a robust and versatile platform for manufacturing multifunctional nanocapsules using physical metal deposition on self-assembled drug-loaded PLGA nanocapsules. Here, we have demonstrated the multifunctionality and synergistic therapeutic effects using three metal–drug–polymer combinations, providing in each of them a proof of concept in a specific relevant application. In the case of iron–PLGA nanocapsules, we demonstrated the capacity to magnetically improve the formation of denser 3D cultures (40% reduction in their cross-section area after 24 h), thus opening the path to a better understanding of the drug efficiency and resistance in an improved representation of a tumor microenvironment.^76^ In the case of gold–PLGA nanocapsules, we showed a dual anticancer photodynamic–photothermal therapy in combination with noninvasive fluorescence imaging with high-penetration NIR light, for enhanced therapeutic effect, showing a 3.5-fold reduction of the tumor volume growth with only 5 min of NIR illumination. With copper–PLGA nanocapsules, we exploited the innate antibacterial properties of copper to improve the antibacterial activity and reduce the dosage of an encapsulated antibiotic drug (a 2.5-fold increase in the MIC50 value), which is a relevant step in the fight against antibiotic resistance.

A central advantage of this approach is the very wide range of metal–drug combinations that can be envisaged to enhance the drug effects and the simple and very accurate control over the thickness and the number of active materials in the structures, without the need of any further fabrication optimization. Therefore, this modular metal–polymer nanocapsule concept provides a high level of versatility that can be exploited to explore new biomedical applications.

## Supporting Information Available

Spheroid (MOV)
Investigation of NCs and silicon surface association regarding thin layer formation (Figure S1) and NC removal (Figure S2); evaluations of NC size, morphology, and metal composition using DLS and SEM/EDS (Figure S3); TEM images of the three representative metal-coated nanocapsules (Figure S4); UV/vis absorbance spectra of the metal-coated nanoparticles (Figure S5); magnetic properties of the Fe-PLGA NCs and magnetic fields used for the formation of the cell spheroids (Figure S6); confocal and SEM images of the Fe-PLGA nanocapsules loaded with rhodamine 6G to demonstrate the internalization of the nanocapsules (Figure S7); measurements of Au-PLGA NC photothermal conversion efficiency (Figure S8); HPLC measurement for the detection of erythromycin (Figure S9); and determination of the release of copper ion from Cu-PLGA NCs (Figure S10) (PDF)

## Supplementary Material

am3c07188_si_001.mov

## Abbreviations

3D: three-dimensional
Au: gold
CFU: colony-forming units
Cu: copper
DDW: double distilled water
DLS: dynamic light scattering
EDS: energy-dispersive X-ray spectroscopy
EE: encapsulation efficiency
Ery: erythromycin
Fe: iron
H&E: hematoxylin and eosin
HPLC: high-performance liquid chromatography
I.T.: intratumoral
ICG: indocyanine green
IVIS: in vivo imaging system
MAPSULES [MPs]: magneto-plasmonic nanocapsules
MDA-MB-231: epithelial breast cancer cell line
MIC: minimum inhibitory concentration
NIR: near-infrared
NPs/NCs: nanoparticles/nanocapsules
NTA: nanoparticle tracking analysis
PDDA: poly(diallyldimethylammonium chloride)
PDI: polydispersity index
PLGA: poly(lactic-*co*-glycolic acid)
ROS: reactive oxygen species
S.C.: subcutaneous
SEM: scanning electron microscopy
SiO2: silicon oxide
TEM: transmission electron microscopy
Ti: titanium
