# Supplementary material for: Modular Drug-Loaded Nanocapsules with Metal Dome Layers as a Platform for Obtaining Synergistic Therapeutic Biological Activities
Source: ACS Appl Mater Interfaces. 2023 Oct 20;15(43):50330–43. doi: 10.1021/acsami.3c07188 (PMC10623511; doi:10.1021/acsami.3c07188)
Supplement: Supplementary file 2 — am3c07188_si_002.pdf [file am3c07188_si_002.pdf]

Supplementary information for

Modular drug-loaded nanocapsules with metal dome layer as platform for obtaining synergistic therapeutic biological activities

Arnon Fluksman[1], Aritz Lafuente[2,3], Ron Braunstein[4], Eliana Steinberg[1], Nethanel Friedman[1], Zhanna Yekhin[5], Alejandro G. Roca[2], Josep Nogues[2,6], Ronen Hazan[4], Borja Sepulveda[7] and Ofra Benny[1*]*

[1] Institute for Drug Research (IDR), School of Pharmacy, Faculty of Medicine, The Hebrew University of Jerusalem, 9112102, Jerusalem, Israel.

[2] Catalan Institute of Nanoscience and Nanotechnology (ICN2), CSIC and BIST, Campus UAB, 08193, Bellaterra, Barcelona, Spain.

[3] Universitat Autònoma de Barcelona, Campus UAB, 08193, Cerdanyola del Vallès, Barcelona, Spain.

[4] Institute of Biomedical and Oral Research (IBOR), Faculty of Dental Medicine, The Hebrew University of Jerusalem, 9112102, Jerusalem, Israel.

[5] Department of Bone Marrow Transplantation and Cancer Immunotherapy, Hadassah Medical Center, The Faculty of Medicine, The Hebrew University of Jerusalem, 9112102, Jerusalem, Israel.

[6] ICREA, Pg. Lluís Companys 23, 08010, Barcelona, Spain

[7] Instituto de Microelectronica de Barcelona (IMB-CNM, CSIC), Campus UAB, 08193, Bellaterra, Barcelona, Spain

* Correspondence should be addressed to O.B. (email: Ofra.Benny@mail.huji.ac.il) and B.S. (email: borja.sepulveda@csic.es)

NCs-silicon surface association

The thin layer formation of the loaded NCs on the silicon wafer, and their removal after the metal evaporation were calibrated to set the conditions to yield as much metal-capped NCs as possible. PAH, PDDA and APTMS were used as positive electrolyte materials at 2% concentrations, in order to provide a positive counter charge to the surface, allowing the negative NCs to attach. The analysis of the SEM images showed that PDDA was the most adequate linker for this purpose (Figure S1). Detachment of the NCs from the surface of the silicon wafer to a 0.2% PSS solution was accomplished using a sonication bath for 5 minutes at 100% sonication power.

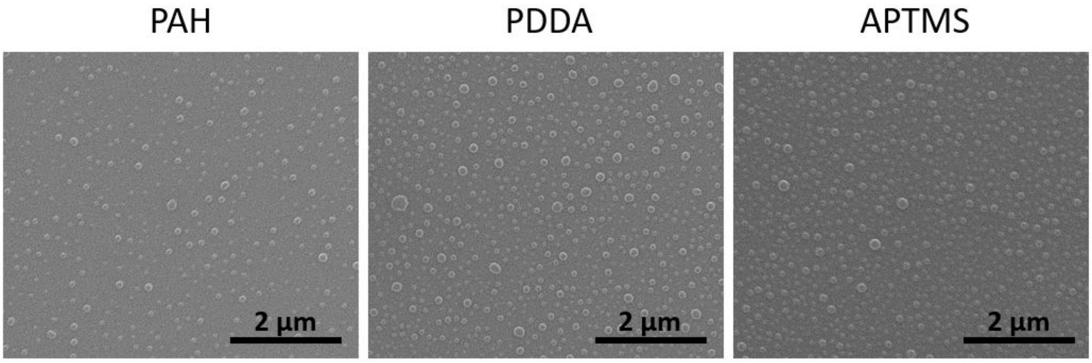

Figure S1. SEM images of the nanocapsules self-assembly on Si wafers precoated with positive monolayers of PAH, PDDA and APTMS at 2% concentration on plasma activated Si wafers.

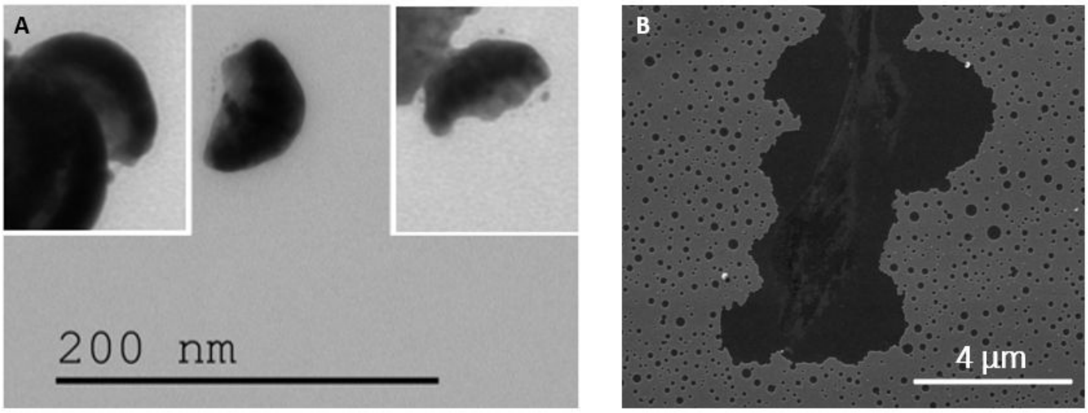

Figure S2. Optimization of metal coated nanocapsules detachment from the wafer. SEM images of the nanoparticles (A) and the substrate (B) for strong sonication conditions, which resulted in metal domes without NPs (A) or damaged wafers (B).

The detachment conditions may vary with the use of different sonication baths; thus, the sonication time and the intensity of sonication must be calibrated accordingly. Figure S2 shows an example of an unsuccessful detachment, when the removal conditions were too aggressive, which resulted in metal domes without the core NP or with damaged wafers.

Morphology and composition characterization using SEM/EDS and DLS

The surface distribution of the particles on the wafer were examined using the environmental Scanning Electron Microscope (SEM) Quanta 200 (FEI Company, The Netherlands). The system includes an energy dispersive X-ray spectroscopy detector (EDS) (EDAX, TSL, AMETEK, Hillsboro, OR, USA) for NC surface elemental identification. Metal-PLGA-covered wafers were cut and fixed by a conductive adhesive tape and placed in a 35 ^{o} tilted platform. Chemical analysis was performed in frame mode, representing the sum of the elemental composition of the imaged NCs and the silicon substrate.

The EDS analysis of the samples confirmed the presence of the different materials in the diverse designed NCs (Figure S3A-C). Figure S3 also shows an example of metal

evaporated NCs loaded on a silicon wafer before (Figure S3D) and after (Figure S3E) sonication using the above-mentioned settings, confirming a high NC recovery from the wafer of ~85%. Finally, the DLS measurements revealed the changes in size distribution of the half coated NCs after the addition of the metal cap, which probably derived from the known drawbacks of the instruments; non-spherical shape of the NCs, different refractive index for the different materials and, false peaks at 10 nm range for NPs larger than 40 nm, as seen previously with the Au-PLGA [1].

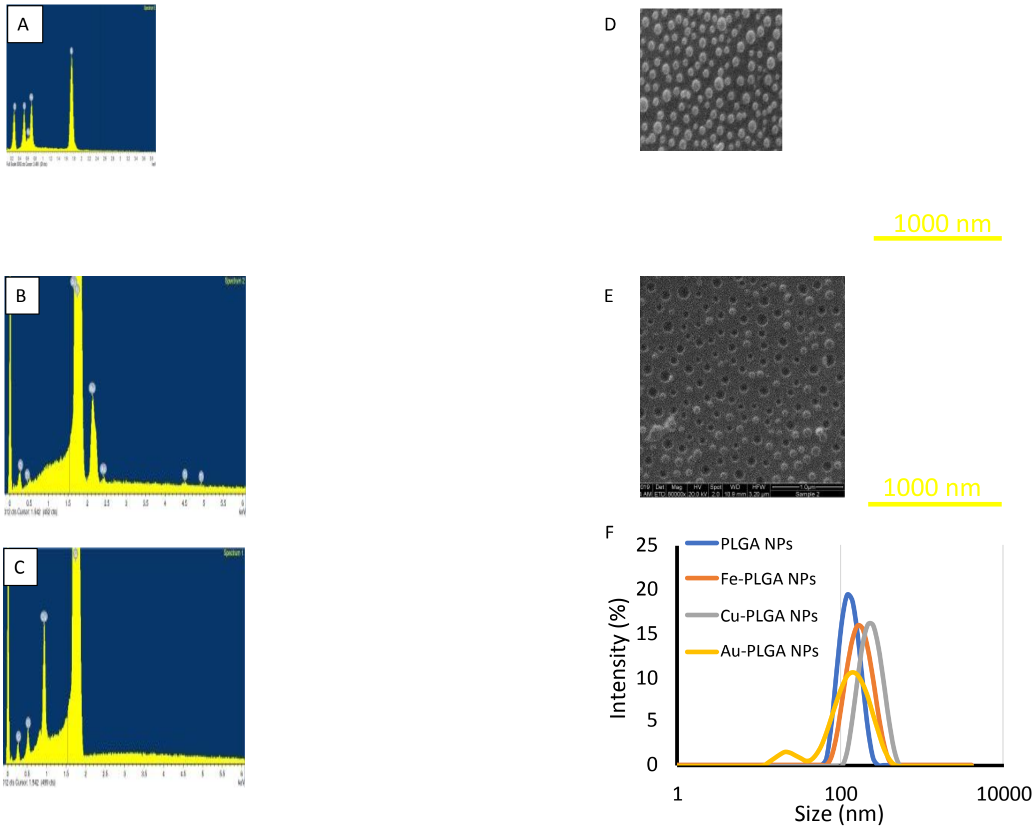

Figure S3. SEM−EDS analysis. EDS elemental spectrum (A-C) from SEM micrographs of Si wafers covered with Fe-PLGA NCs (A), Au-PLGA NCs (B) and Cu-PLGA NCs (C). SEM micrographs of a wafer before (D) and after (E) sonication. (F) DLS intensity-based size distribution evaluation of Metal-PLGA NCs after removal.

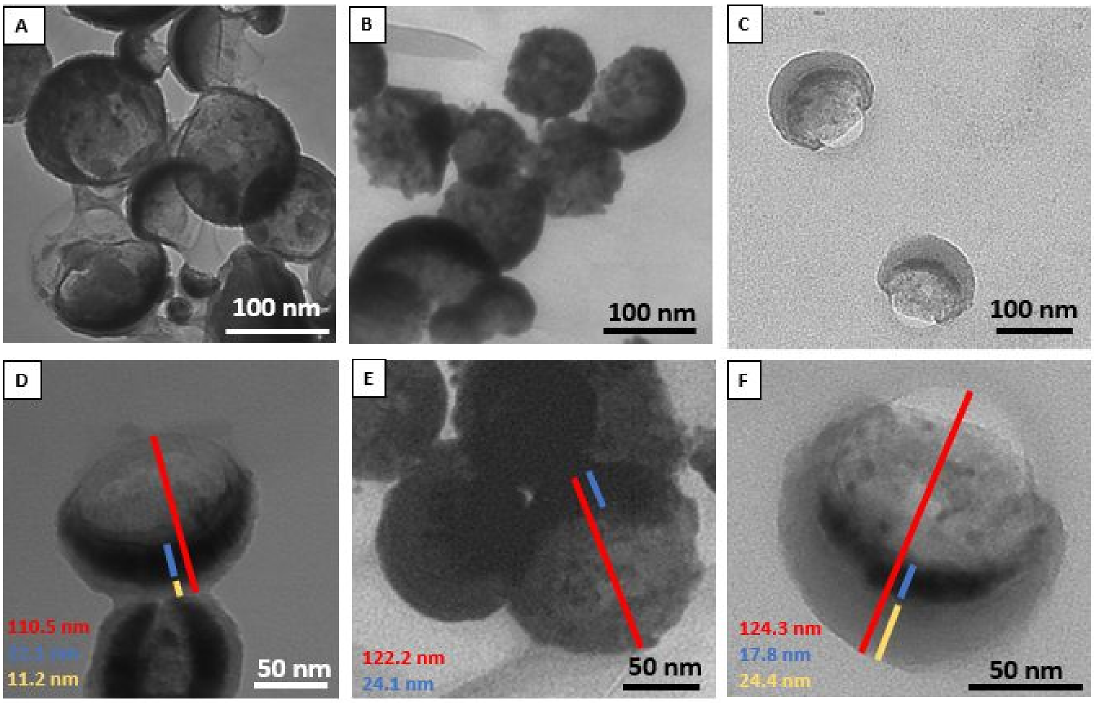

Figure S4. Nanoparticle morphology. TEM micrographs and separation into layers of Fe-PLGA NPs (A and D), Au-PLGA NPs (B and E) and Cu-PLGA NPs (C and F). Red- whole NP, Blue- first layer and yellow- second layer.

UV/vis absorbance spectrum of NCs

Beside the morphology, composition, size and charge of the NCs, the UV/Vis absorption spectra were recorded using a spectrophotometer (FluoroMax4, HORIBA, USA). NCs were recovered from the wafers as depicted above and counted. Samples vials were photographed and 600 μl were loaded to a quartz vial for UV/vis absorbance spectrum measurement of each metal capped NCs, recorded in the range of 270-870 nm.

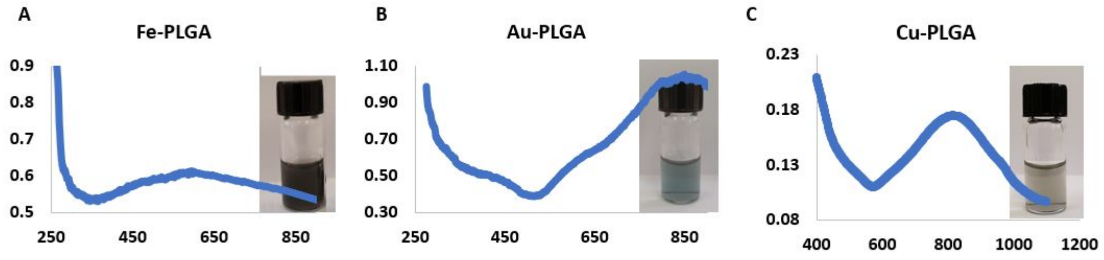

Figure S5. UV/vis absorbance spectrum of Fe-PLGA NCs (A), Au-PLGA NCs (B) and Cu-PLGA NCs (C) and images of the NCs solutions accordingly.

Magnetic properties and magnetic manipulation of the Fe-PLGA nanoparticles

Figure S6a shows the SQUID magnetometry magnetization loops of the Fe-PLGA nanoparticles. The magnetic properties of the nanocapsules were recorded using a SQUID magnetometer (MPMS-XL, Quantum Design). A magnetization curve at 300 K with a maximum applied field of 50 kOe was performed on tightly packed powdered sample after drying some drops of the nanodome suspension in water.

Note the loops show virtually zero magnetization in the absence of magnetic field. In fact, the constriction of the hysteresis loop at low fields is typical of a vortex magnetization reversal.[2] Moreover, it is important to highlight the field required to saturate the magnetization is only about 2 kOe.

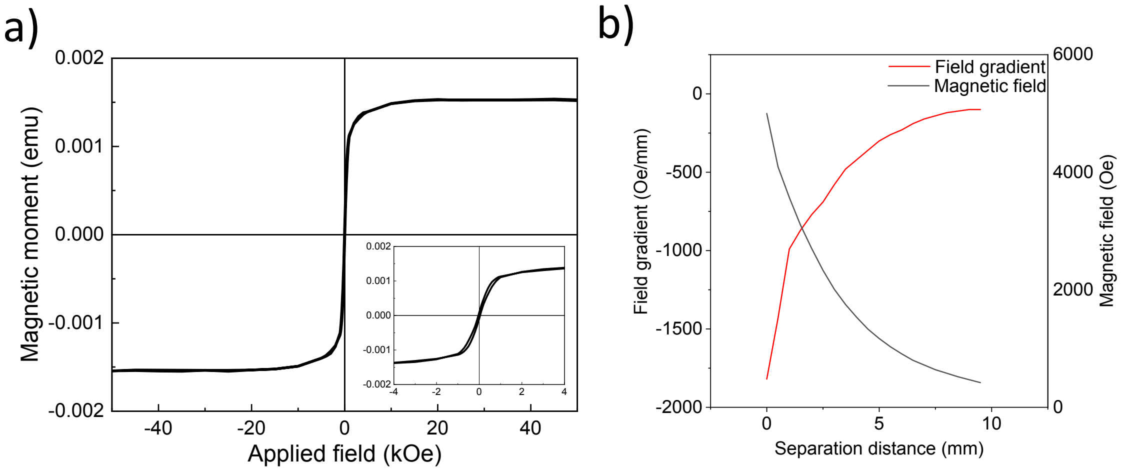

Figure S6. a) SQUID magnetometry magnetization reversal loop of the Fe-PLGA nanoparticles. The inset shows the magnetic behavior at low field. b) Measurement of the magnetic field and field gradient of the cylindric permanent magnet used for the formation of the cancer cell spheroids.

In vitro fluorescence and SEM assay to determine the internalization of the Fe-PLGA nanoparticles.

Confocal Scanning Laser Microscopy (CSLM) images were taken using Leica SP5 (Leica microsystems)

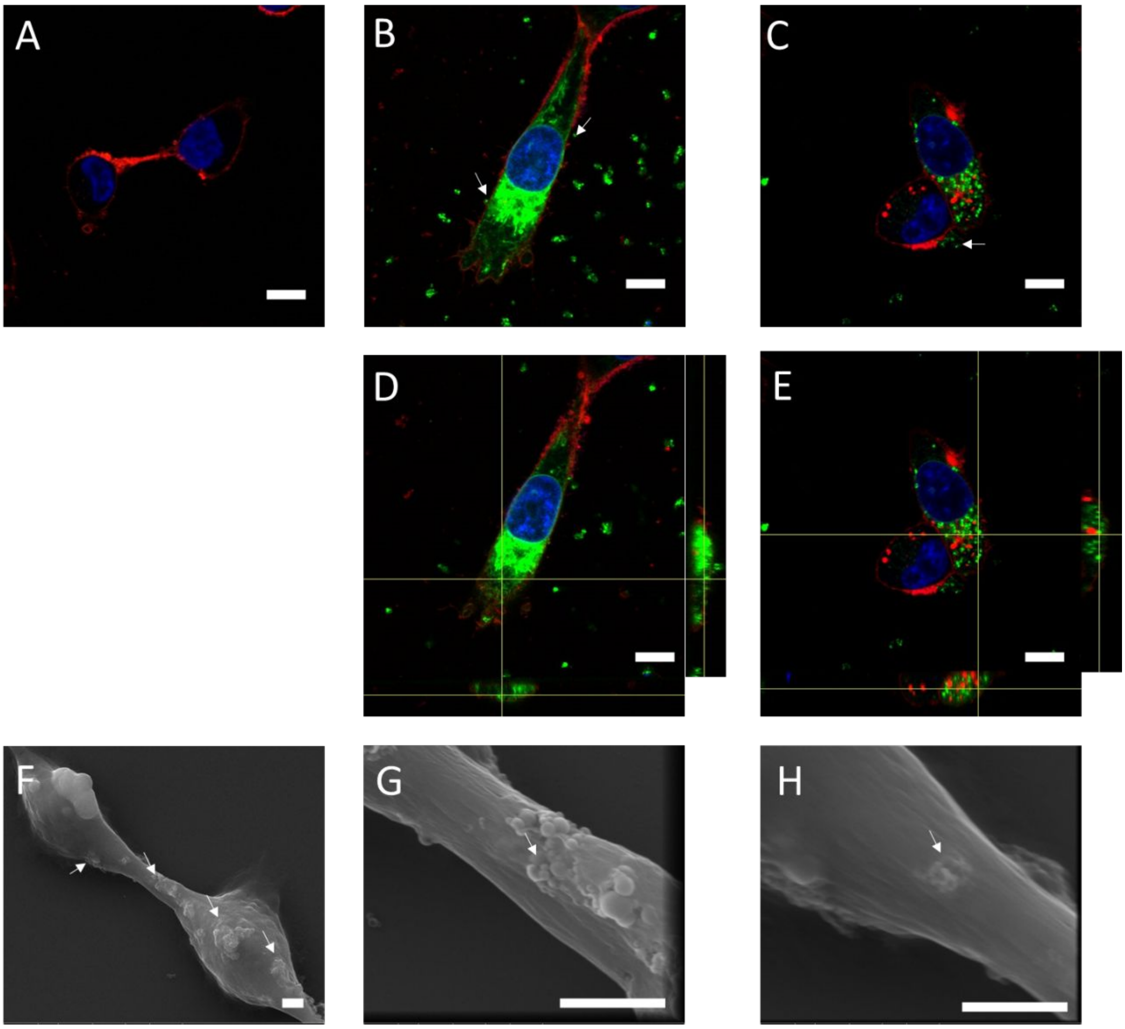

Figure S7. Images of the Fe-PLGA nanoparticles interacting with the MDA-MB-231 cells. CLSM fluorescence images of (A) Control without particles, after incubation with rhodamine loaded Fe-PLGA nanoparticles for (B) 4h and (C) 24h respectively. White

arrows show the interaction of particles with the membrane. Orthogonal views showing the internalization of particles at (D) 4h and (E) 24h respectively. Scale bar 10 µm. (F-H) SEM micrographs showing the interaction of Fe-PLGA nanoparticles with cell membrane after 4h of incubation. White arrows show the interaction of particles with the membrane Scale bar 2 µm.

Photothermal heating efficiency measurement in Au-PLGA nanocapsules

The photothermal conversion efficiency of the NCs different nanocapsules was measured using a specially designed photothermal testing system. The system consisted of a NIR laser diode with emission wavelength of 808 nm (L808P500MM, Thorlabs) regulated by a laser diode controller (ITC4005, Thorlabs) and a power meter (PM100D, Thorlabs). Temperature monitored by an infrared camera (ETS320, FLIR).

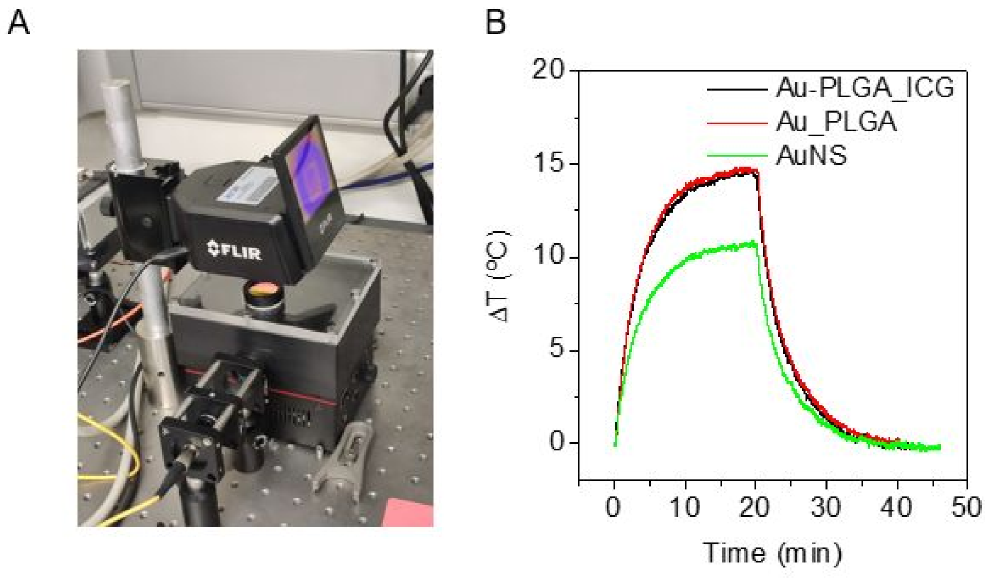

Figure S8. Au-PLGA photothermal conversion efficiency. (A) experimental set-up. (B) Temperature elevation of commercial nanoshells, ICG loaded and unloaded Au-PLGA nanoparticles irradiating with 808 nm laser, 267 mW (1 W/cm^2) for 20 minutes.

Erythromycin detection by HPLC.

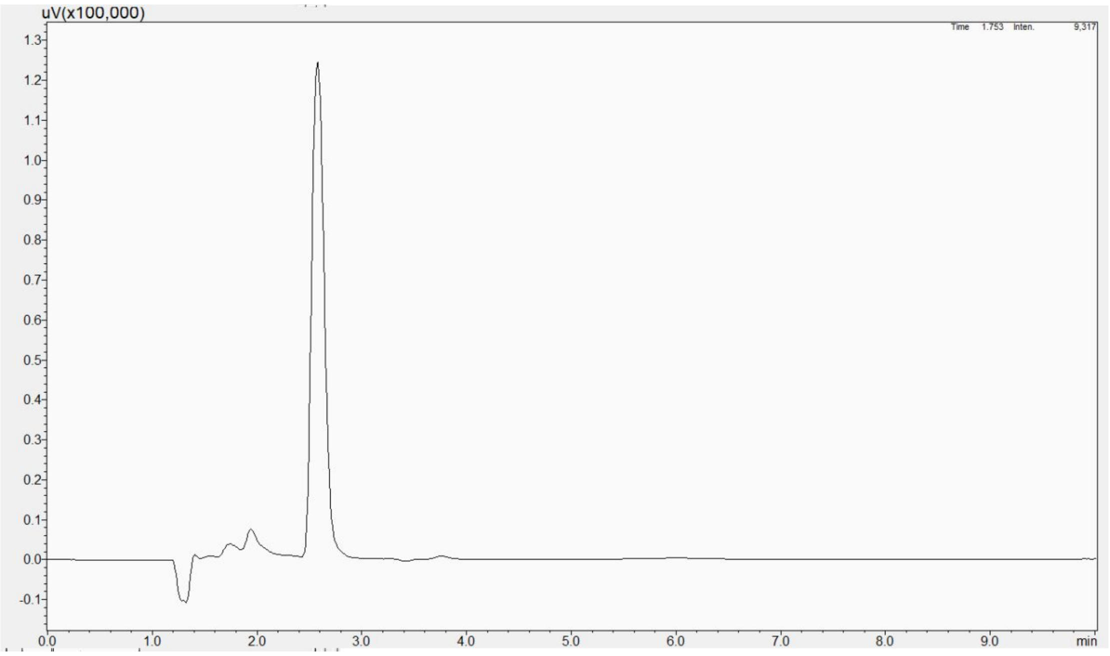

Figure S9. Chromatogram of erythromycin in acetonitrile.

Copper ion release from Cu-PLGA NCs

The release of copper ion was demonstrated using a spectrophotometer to measure the UV/Vis absorption spectra of Cu-PLGA NC solutions before and after 24 hour incubation at 37 ^{o}C. The significant drop in absorbance, also visible with the naked eye where the solution became transparent, demonstrates the extensive copper ion release from the copper cap. To evaluate the extent of release, solutions were centrifuged at 14,000 rpm for 10 min and the supernatant and pellet were separated for ICP-OES measurements (Perking Elmer Optima 4300DV). The Cu ion release from the Cu-PLGA NCs to the medium was 77% from initial Cu content.

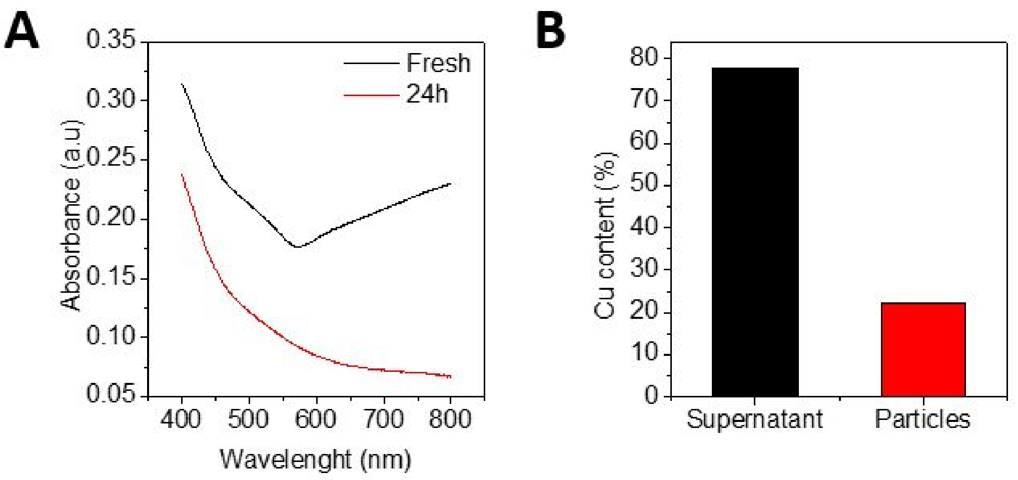

Figure S10. Evolution of Cu-PLGA NCs with time. (A) Evolution of the absorbance of Cu-PLGA NCs incubated at 37 °C. (B) Determination the fraction of copper content in the nanocapsules and supernatant after 24h, measured by ICP-OES.

References

(1) Khlebtsov, B. N.; Khlebtsov, N. G. On the Measurement of Gold Nanoparticle Sizes by the Dynamic Light Scattering Method. *Colloid Journal* **2011**, *73* (1), 118–127. https://doi.org/10.1134/S1061933X11010078.

(2) Li, Z.; Aranda-Ramos, A.; Güell-Grau, P.; Tajada, J. L.; Pou-Macayo, L.; Lope Piedrafita, S.; Pi, F.; G. Roca, A.; Baró, M. D.; Sort, J.; Nogués, C.; Nogués, J.; Sepúlveda, B. Magnetically Amplified Photothermal Therapies and Multimodal Imaging with Magneto-Plasmonic Nanodomes. *Appl Mater Today* **2018**, *12*, 430–440. https://doi.org/10.1016/j.apmt.2018.07.008.
